# Spectral Analysis and Antiulcer Potential of *Lactuca sativa* through the Amelioration of Proinflammatory Cytokines and Apoptosis Markers

**DOI:** 10.3390/life12101641

**Published:** 2022-10-19

**Authors:** Rahamat Unissa Syed, Sivakumar S. Moni, Amr S. Abu Lila, Marwa H. Abdallah, Amr S. Abouzied, Humera Banu, Khetam Saad Mutni Alreshidi, Badriah Mansour Wadid Alrashidi, Mohd Abdul Hadi, Hemat El-Horany, Siddig Ibrahim Abdelwahab, Manal Mohamed Elhassan Taha

**Affiliations:** 1 Department of Pharmaceutics, College of Pharmacy, University of Hail, Hail 81442, Saudi Arabia; 2 Department of Pharmaceutics, College of Pharmacy, Jazan University, Jazan 45142, Saudi Arabia; 3 Department of Pharmaceutics and Industrial Pharmacy, Faculty of Pharmacy, Zagazig University, Zagazig 44519, Egypt; 4 Department of Pharmaceutical Chemistry, College of Pharmacy, University of Hail, Hail 81442, Saudi Arabia; 5 Department of Pharmaceutical Chemistry, National Organization for Drug Control and Research, Giza 12553, Egypt; 6 Department of Clinical Nutrition, College of Applied Medical Sciences, University of Hail, Hail 81442, Saudi Arabia; 7 College of Pharmacy, University of Hail, Hail 81442, Saudi Arabia; 8 Department of Pharmaceutics, School of Pharmacy, College of Health Sciences and Medicine, Wolaita Sodo University, Wolaita Sodo 46551, Ethiopia; 9 Department of Biochemistry, College of Medicine, University of Hail, Hail 81442, Saudi Arabia; 10 Department of Medical Biochemistry, Faculty of Medicine, Tanta University, Tanta 31511, Egypt; 11 Medical Research Center, Jazan University, Jazan 45142, Saudi Arabia; 12 Substance Abuse and Toxicology Research Centre, Jazan University, Jazan 45142, Saudi Arabia

**Keywords:** bioactive compounds, spectral analysis, anti-ulcer activity

## Abstract

The objective of this study was to characterize the bioactive ingredients and antiulcer effects of *Lactuca sativa leaves*. Several bioactive chemicals were found in the cold methanolic extract of *Lactuca sativa* leaves after gas chromatography-mass spectrometry (GC-MS) research: 9,12-octadecadienoic acid (Z,Z)-, cyclononasiloxane, octadecamethyl-, n-hexadecanoic acid, Hexadecanoic acid, 2-hydroxy-1-(hydroxymethyl)ethyl, octadecanoic acid, 2-hydroxy-1-(hydroxymethyl)ethyl ester, 9-octadecenamide, (Z)-, hexadecanoic acid, stigmasterol, benzothiazole, ethyl iso-allocholate, and octacosane. Distinct fingerprint regions in GCMS indicated the existence of bioactive compounds. The leaf powder of *Lactuca sativa* (LPL) demonstrated substantial antiulcer properties at 400 mg/kg, which was almost equivalent to the standard drug at 20 mg/kg. The cytokine network was efficiently regulated by reducing the production of proinflammatory cytokines such as IL-1β, IL-6, and TNF-α. The levels of caspase-3 and caspase-9 were also considerably lowered at *p* < 0.05 significant level.

## 1. Introduction

Disease prevention has traditionally been regarded as preferable to treating the disease using various therapeutic approaches. Food is the most fundamental requirement for humans because it provides both mental and physical energy. Aside from significant nutrient components, all foods have a suitable number of functional features that help one’s health directly or indirectly.

According to the findings of several different scientific studies, eating certain types of vegetables, fruits, and grains may help prevent or delay the beginning of certain diseases, and this practice should be prioritized for the benefit of human society. The use of specific culinary herbs to prevent the development of particular ailments will be necessary for the welfare of human society [1]. *Lactuca sativa Linn.*, also known as lettuce, is renowned for its use in the preparation of food, particularly salads [2]. Additionally, this plant possesses remarkable qualities for use in medicine. Due to its delicious flavor and excellent nutritional content, lettuce is grown worldwide and is one of the green leafy vegetables most widely consumed in its raw state [2,3]. Earlier studies suggested *Lactuca sativa* has antioxidant, hypoglycemic, sedative, hypnotic, analgesic, and anticonvulsant properties [4,5,6]. In addition, an earlier study showed that lettuce plants are adequate as a bioindicator in environmental investigations [7].

Ulcers of the stomach are one of the most prevalent disorders that can affect the digestive system. The pathophysiology of gastric ulcers is an imbalance between gastric mucosa-protecting and gastric mucosa-destroying due to acid secretions. Infectious diseases, smoking, stress, prolonged use of steroidal as well nonsteroidal antiinflammatory medicines, and excessive alcohol intake are also contributing factors that produce this imbalance [8]. Herbal medicines are alternative source of medicine that can cure a variety of gastroprotective mechanisms, such as the promotion of mucosal proliferation, suppression of acid, wound healing characteristics, production, and antioxidant capabilities [8]. The present study focused on demonstrating the chemical characterization through cold methanolic extraction and the ulcer healing properties of *Lactuca sativa* (LPL) leaf powder.

## 2. Materials and Methods

### 2.1. Collection and Processing

*L. Sativa* leaves were purchased in bulk from the local market of the Hail region, Saudi Arabia. The leaves were immediately transferred into sterile polyethylene bags and transported to the laboratory. Adhered impurities were removed by rinsing the leaves under running tap water, followed by millipore water. Leaves were then shade-dried for seven days. The specimens of washed leaves were authenticated and deposited in the Hail University herbarium (UOHCOP012). The air-dried sample was finely powdered and stored in a sealed container. The resultant sample powder was assigned as LPL.

### 2.2. Materials

The solvents and chemicals needed for the current study were bought from Sigma (St. Louis, MO, USA). All items were supplied by Ejadah Medical Supplies Est., Riyadh, K.S.A.

### 2.3. Extraction Procedure

Bioactive parts of the LPL were extracted using the cold methanol maceration method. The reaction mixture (RM) was developed by soaking 10 g of LPL in 10 mL of methanol and mixed with the help of a magnetic stirrer for 90 min at room temperature. The RM was refrigerated overnight at a temperature of 4 °C. This method was repeated for one additional week. The macerated reaction mixture was centrifuged with a TGL-16 benchtop centrifuge at 2000× *g* for 10 min. The supernatant solution was then filtered through Whatman filter paper (No. 1) and dried at room temperature for developing the extract. The resultant sample was stored at 4 °C for further use [9].

### 2.4. GC-MS Analysis

The GC–MS chromatogram of the LPL extract was carried out using the Thermo Scientific GC-MS 3000 autosampler (ISQ detector); this equipment was employed for the determination of bioactive compounds. The carrier gas was 99.99% pure helium gas, and the flow rate was maintained at 1 mL/min. The injector was run at 250 °C, whereas the temperature of the oven was maintained at 60 °C for 15 min and then gradually increased to 280 °C for 2 min. The MS data was collected between 30 to 600 m/z with a 2 min solvent cutoff. The data were assembled and processed using X Calibur software. The interpretation of the mass spectra was performed using the MAINLIB and NIST software libraries [10].

### 2.5. FT-IR Spectroscopy

The FT-IR was performed with a resolution in the spectral area of 4000–400 cm^−1^ to detect the possible functional groups. A total of 10 mg of LPL was encapsulated in 100 mg of KBr salt pellets using a mortar and pestle, compressed into a thin pellet. FT-IR spectra was investigated using the Nicolet iS10 FT-IR spectrophotometer, Thermo Scientific (Waltham, MA, USA) [9,11].

### 2.6. In Vivo Study

#### 2.6.1. Experimental Animals

Male Wistar rats weighing about 150 ± 30 g were procured from the Medical Research Centre (MRC), Jazan University. Before beginning the study, the animals were quarantined for two weeks at standard laboratory conditions (22 ± 0.8 °C and a relative humidity of 56 ± 6%). The animals were provided with clean water and food.

#### 2.6.2. Gastric Ulcer Model

Twenty animals were uniformly distributed into four groups, with five of them in each group. The studies were performed according to Al-Wajeeh, N.S. et al., 2016. [12].

Group 1: Normal control group: The animals did not undergo any treatment. 

Group 2: Disease control group: Ulceration group: Ulcers were formed by introducing 95% (*v*/*v*) ethanol (5 mL/kg body weight).

Group 3: Standard drug treatment group: These animals received omeprazole (20 mg/kg body weight in distilled H_2_O) as a single oral dose at 2 h before ethanol introduction. 

Group 4: Test drug treatment group: These animals received LPL at an oral dose of 400 mg/kg body weight (predetermined concentration) in distilled H_2_O as a single dose 2 h before administration of 95% (*v*/*v*) ethanol.

#### 2.6.3. Determination of Ulcer Index and % Inhibition of Ulcer [13]


(1) $$U.I,=\frac{Ulceration\;area}{Total\;stomach\;area}\times 100$$

The % inhibition of ulceration was calculated as follows:

(2) $$\%\;Inhibition=\frac{\left(Ulcer\;index\;of\;control\right)\;-\;\left(Ulcer\;index\;of\;the\;test\right)}{Ulcer\;index\;of\;control}$$

#### 2.6.4. Macroscopic and Biochemical Gastric Assessments

The tissues of the stomach were separated for macroscopic and pathological analysis. The images were captured with the use of a USB digital microscope furnished with an endoscopic camera. The percentage inhibition was calculated using a moderate improvement of the standard set forth in a previous publication, and the entire ulcerated area was assessed utilizing the standard methodology with minor alterations [14]. The findings of the pH meter and sodium hydroxide solution titration used to measure acidity are displayed in milliequivalents per liter [15]. A sensitive digital balance was used to find mucus weight. Animal tissues and stomach contents were separated for microscopic and pathological examination. According to previous study, the total ulcerated area and the percentage inhibition were calculated [14].

#### 2.6.5. Collection of Serum

The animals’ tail veins were used to draw blood the next day. Without using an anticoagulant, the collected blood was pooled and kept in separate Flacon blood collection tubes. Slanting the tubes and centrifuging the blood samples at 2000× *g* for 10 min in a refrigerator separated the serum from the blood samples. The serum was recovered by centrifuging the supernatant, which was then collected and kept in a refrigerator between 2 and 8 °C. The serum was fractionated from the blood samples by resting the tubes in an inclined position and was centrifuged at 2000× *g* for 10 min in a refrigerated centrifuge. The serum was tested the following day using an enzyme-linked immunosorbent assay (ELISA) to measure the levels of the proinflammatory cytokine interleukin-1β (IL-1β), interleukin-6 (IL-6), and tumor necrosis factor (TNF-). The apoptosis markers caspase-9 and caspase-3 were also identified by ELISA [16]. Briefly.

##### Serum IL-1β

The rat IL-1β ELISA kit from MyBioSources (San Diego, CA, USA), was used to quantitatively assess the quantity of IL-1β in the serum. The presence of IL-1β in the serum was determined using a sandwich ELISA, which was read at 450 nm immediately using an ELISA reader, ELx800, USA. The concentration of IL-1β was calculated by extrapolating on the standard curve and was expressed in pg/mL.

##### Serum IL-6

The rat IL-6 ELISA kit, MyBiosource (San Diego, CA, USA), was used to quantitatively quantify the serum interleukin-6 (IL-6) level. The assay uses a double antibody sandwich approach and is based on the properties of a target analyte with many potential epitopes that can be concurrently recognized by the precoated capture antibody and the detection antibody. The end point was determined by reading the ELISA plate at 450 nm immediately using an ELISA reader, ELx800, USA. The concentration of IL-6 was calculated by extrapolating on the standard curve and expressed in pg/mL.

##### Serum TNF-α

The serum TNF-α concentration was quantified using a rat TNF-α ELISA kit, MyBiosource, USA. The kit employs a sandwich enzyme immunoassay for the in vitro detection and quantification of TNF- α in rat serum. The end point was determined by reading the ELISA plate at 450 nm immediately using ELISA reader, ELx800, USA. The quantity of TNF-α was found by extrapolating on the standard curve and was expressed in pg/mL.

##### Caspase-3 and -9

The serum levels of caspase-3 and 9 were quantified using the rat caspase-3 and caspase-9 ELISA kit, MyBiosource (San Diego, CA, USA). The kit employs a double-antibody sandwich enzyme immunoassay for quantitative in vitro detection of caspase-3 and 9 in rat serum. The end point was determined by reading the ELISA plate at 450 nm immediately using an ELISA reader, ELx800, USA. The concentrations of caspase-3 and 9 were determined by extrapolating on the standard curve and were expressed in ng/mL.

### 2.7. Statistical Analysis

Data are presented as mean ± SD. Comparison amongst groups was done with ANOVA and Dunnett’s multicomparison test. Values of *p* < 0.05 indicated statistically significant differences. Statistical analysis was performed by the GraphPad Prism, 9 software, (San Diego, CA, USA).

## 3. Results and Discussion

**Figure 1:**
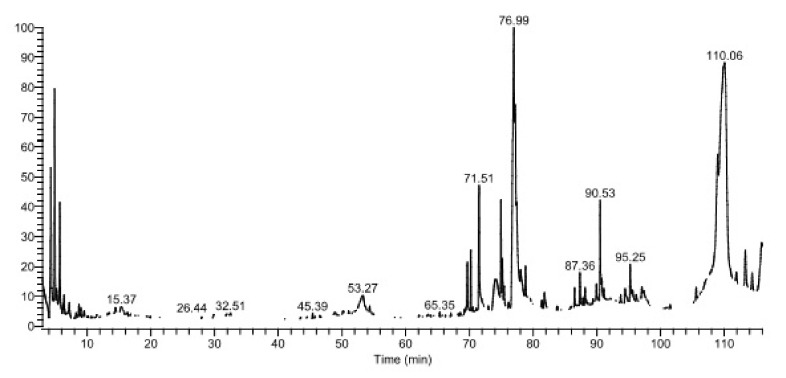
GC-MS chromatogram of the cold methanolic extract of leaf powder of *Lactuca sativa*.

**Figure 2:**
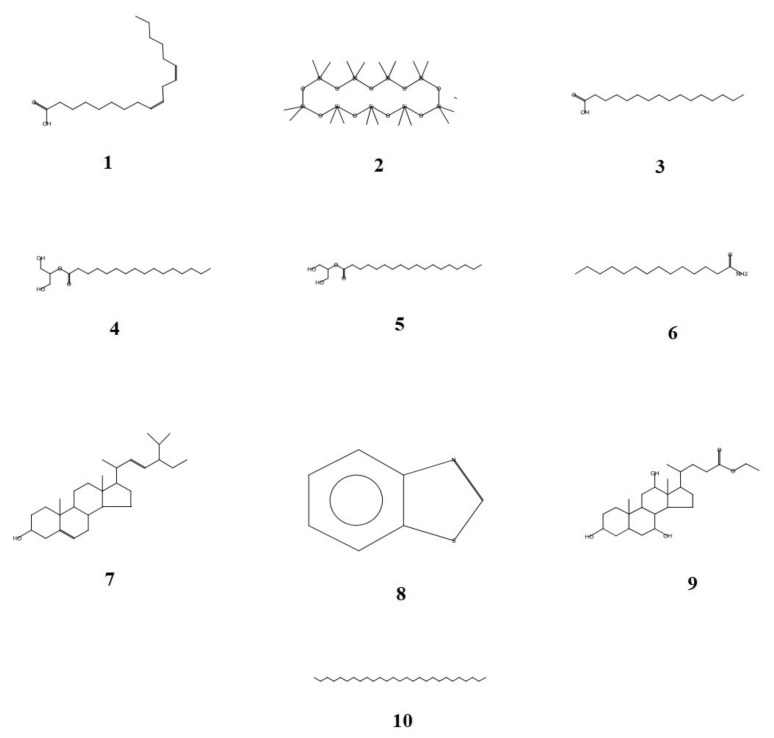
GC-MS detection of possible bioactive compounds of the cold methanolic extract of leaves powder of *Lactuca sativa* (**1**) 9,12-Octadecadienoic acid (Z,Z)- (**2**) Cyclononasiloxane, octadecamethyl- (**3**) n-Hexadecanoic acid (**4**) Hexadecanoic acid, 2-hydroxy-1-(hydroxymethyl)ethyl (**5**) Octadecanoic acid, 2-hydroxy-1-(hydroxymethyl)ethyl ester (**6**) Octadecanamide (**7**) Stigmasterol (**8**) Benzothiazole (**9**) Ethyl iso-allocholate (**10**) Octacosane.

Herbs are a distinctive and significant source of bioactive compounds, which exist in various molecular configurations and may have medicinal benefits. Plants are used in traditional medicine to treat a wide range of disorders because they contain many biologically active compounds with therapeutic potential. Vegetables are healthy because they contain vitamins, minerals, phytochemicals, and dietary fiber. An adequate vegetable diet has been found to reduce the risk factors linked with various chronic diseases, such as cardiovascular diseases, metabolic syndrome, diabetes, obesity, and cancer [17]. As depicted by the chromatogram in Figure 1, GC-MS analysis confirmed the existence of many bioactive chemicals. Table 1 depicts the existence of several bioactive components, and Figure 2 illustrates their structures. 

In the GC-MS chromatogram, 9,12-octadecadienoic acid (Z,Z), also known as α-linoleic acid, demonstrated a retention time (RT) of 76.09 min with a probability of 55.92% and occupied the maximum, nearly 16.48%. According to a previous investigation, linoleic acid was found in the petroleum ether extracts of the leaves of *L. sativa.* This acid was present at 57.05 min and occupied 3.24% in the chromatogram [18]. According to another study, linoleic acid was the most abundant component in the seeds of *L. tatarica* [19]. An earlier study suggested that conjugated linoleic acids exhibited a strong antioxidant effect and showed an anticarcinogenic effect [20,21]. The conjugated linoleic acid (LA) also proved to have antiobesogenic and antiatherosclerotic effects [22]. α- L A reduces the risk of heart disease by restoring normal heart rhythm and lowering blood clotting and pumping motion, according to certain research [23,24,25,26,27]. Cyclononasiloxane octadecamethyl was observed at a maximum RT of 110.08 min with 22.9% of probability index and occupied 23.76% in the chromatogram. An earlier report suggested that cyclononasiloxane octadecamethyl has good antifungal properties [28]. N-hexadecanoic acid, also known as palmitic acid (PA), was discovered at 71.51 min RT with a probability index of 72.04% and occupied 3.76% in the chromatogram. The antiinflammatory effects of PA were reported in the previous studies. Moreover, it demonstrated a potent antibacterial effect on biofilm-forming bacteria [29,30]. Hexane extract of *Lactuca serriola* showed the presence of palmitic acid, detected at 28.45 min of RT [31]. 

**Figure 3:**
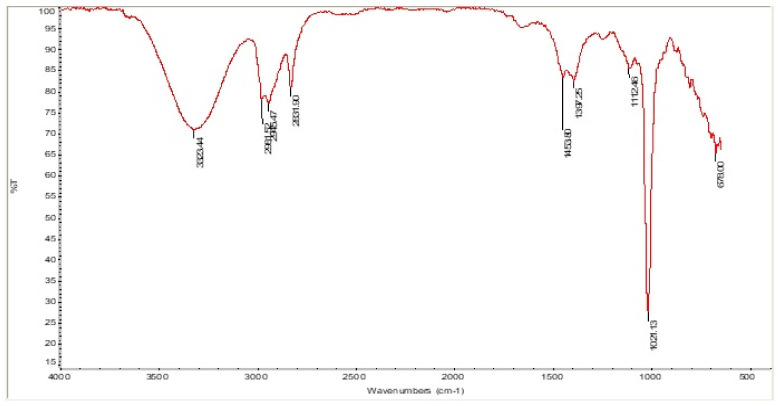
The FT-IR spectra of compounds of the cold methanolic extract of leaves powder of *Lactuca sativa*.

A recent investigation suggested that palmitic acid has a strong inhibitory effect on the proliferation of prostate cancer cells both in vitro and in vivo [32]. Previous studies have demonstrated that PA can inhibit the growth of various pathogens, including bacteria and fungi [33]. Hexadecanoic acid, 2-hydroxy-1-(hydroxymethyl)ethyl ester, also known as palmitic acid-monoglyceride, appeared at 90.53 min RT with a probability index of 61.55 and occupied 2.69% of the chromatogram’s area. β-PA is a natural saturated fatty acid seen in human milk. It affects the metabolism of fatty acids, boosts mineral balance, enhances infants’ sleep patterns, and reduces crying [34,35]. Octadecanoic acid, 2-hydroxy-1-(hydroxymethyl)ethyl ester, a fatty acid otherwise called glycerol monostearate, and β-Monostearin were detected at 95.25 min RT with 63.34 probability index and occupied 1.37% in the area in chromatogram. Glycerol monostearate is a preservative: with an anticaking, emulsifying, and thickening agent in foods; as an emulsifier for solvents, oils and waxes; as a protective coating for hygroscopic powders; and as a solidifier and control release agent in pharmaceuticals [36]. Octadecanamide is a fatty amide of stearic acid which has been detected at 87.36 min RT with 87% of probability index and occupied 0.78% in the chromatogram. Followed by stigmasterol, it was eluted at 111.90 min RT with 71.04% of probability index and occupied only 0.38% of the chromatogram. According to an earlier report, stigmasterol is a powerful antiosteoarthritic substance that blocks proinflammatory mediators [37]. Earlier reports suggested that stigmasterol exhibited mild antiulcer properties [38]. Benzothiazole, ethyl iso-allocholate, and octacosane have been detected as trace elements in the methanolic extract of LPL (Table 1). The FT-IR analysis revealed the existence of distinctive peaks with significant functional groups consistent with bioactive substances (Figure 3). The sharp parabola-shaped peak at 3323.44 cm^−1^ with stretching vibrations suggests the presence of phenolic O–H groups, which correlate to the phenolic OH group corresponding to steroids. The present study also exhibited distinct peaks at 2981.52, 2945.47, and 2831.90 cm^−1^, with stretching vibrations corresponding to the presence of fatty acids, aliphatic compounds, and steroids. The peaks at 1453.80, 1112.46, and 1021.13 cm^−1^ also indicate the occurrence of fatty acids, alkanes, and carbohydrates. 

**Figure 4:**
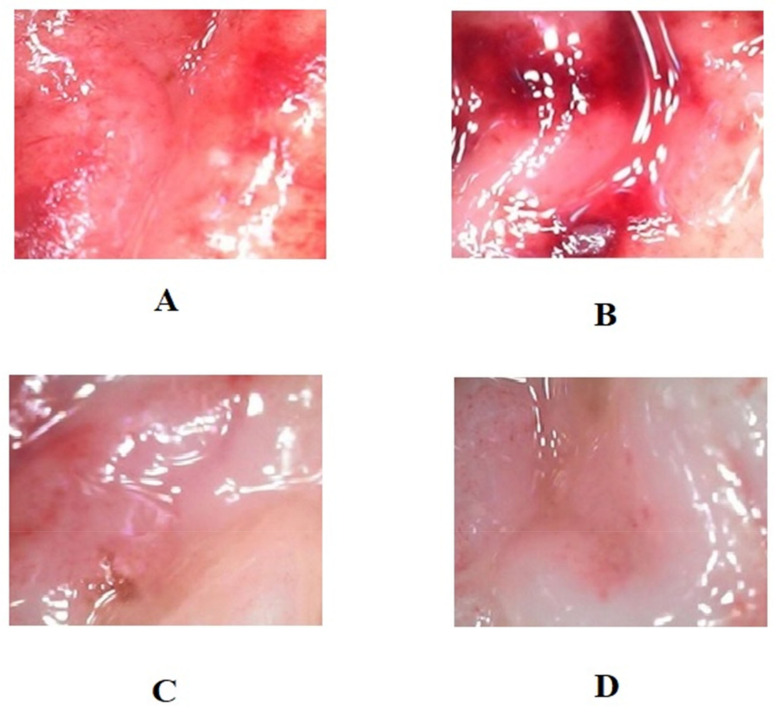
Macroscopic inspection of hemorrhagic lesions present on the glandular part of the rat stomach. (**A**–**D**) are representative photos from groups 1, 2, 3, and 4, respectively.

**Figure 5:**
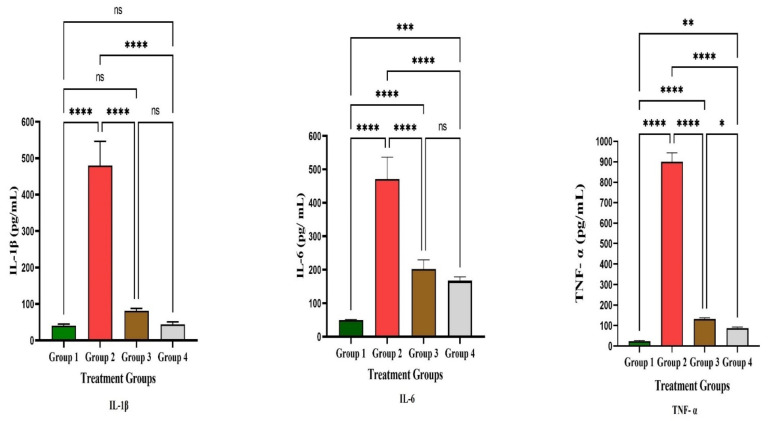
Study on proinflammatory cytokines. **** The values are very highly significant at *p *< 0.05 level. *** The values are highly significant at *p* < 0.05 level. ** significant at *p *< 0.05 level, * significant at *p *< 0.05 level; ns: nonsignificant at *p* < 0.05 level.

**Figure 6:**
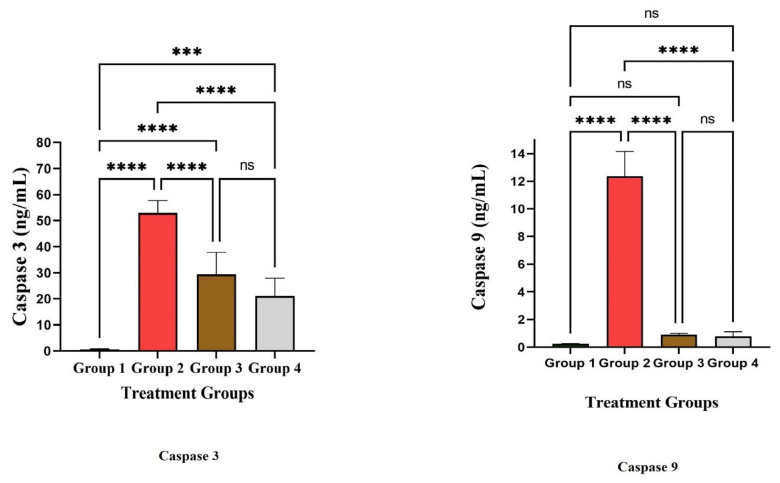
Study of apoptosis markers. **** The values are very highly significant at *p *< 0.05 level. *** The values are highly significant at *p *< 0.05 level. ns: nonsignificant at *p *< 0.05 level.

Table 2 shows the impacts of LPL and omeprazole on the treatment of gastric ulcers in Wistar rats. The macroscopically depicted hemorrhagic lesions on the glandular portion of the rat stomach are shown in Figure 4. According to the results, the measured ulcer area of animals in the control group was 611 ± 32 mm^2^. When compared to the control group, the ulcer area of the groups subjected to treatment with LPL at a dose of 400 mg/kg body weight was significantly reduced, measuring 90 ± 3.5mm^2^, indicating 74 ± 1.9% inhibition. However, omeprazole treatment resulted in an ulcer area of 90.5 ± 10.58 mm^2^, i.e., 85 ± 1.9% healing rate. In the ulcer control group, the therapeutic efficacy of LPL at a dose of 400 mg/kg body weight exhibited a similar effect with 85.18 ± 2.71% inhibition. Macroscopic examination demonstrated that the LPL pretreated group and the omeprazole pre-treated group had considerably less stomach damage than the ulcer control group (Figure 4). There were marked increases in IL-1β, IL-6, and TNF-α in group 2 after the induction of ulcers (Figure 5). However, these factors’ levels decreased significantly in the treatment groups (groups 3 and 4; *p* < 0.05). An earlier study suggested that the effects of lettuce consumption with a moderately high-fat meal did not differ in the plasma IL-6 and TNF-α concentrations [39]. In the present study, proinflammatory cytokine levels were modulated due to the presence of α linoleic acid. The apoptosis marker caspases-3 and -9 were increased markedly in group 2 after the induction of ulcers (Figure 6). The results showed that both caspase-3 and caspase-9 were significantly reduced when compared to the disease group 2. From this study, it is evident that LPL exerted immunomodulatory properties, which led to the cure of ulcers.

## 4. Conclusions

Plants have long been used as good sources for the evolution of medicinal agents. Consuming a diet rich in fruits and vegetables is advantageous to maintaining good health. The pharmaceutical significance of lettuce has not been adequately explored, although lettuce is rich in biochemicals that aid in the prevention of several diseases. The present investigation investigated the bioactive constituents of *Lactuca sativa* (lettuce) using a cold methanolic extract. The study proved the efficacy of ingesting raw *Lactuca sativa* leaves in preventing stomach ulcers through the modification of proinflammatory cytokines and apoptotic indicators. The results of this study are extremely encouraging for the development of innovative antiulcer medication molecules, which will be a substantial contribution to the advancement of human welfare. In addition, the study recommends that ulcer sufferers consume lettuce leaves to aid in natural recovery.

## Figures and Tables

**Table 1:** Possible bioactive compounds detected in GCMS of *Lactuca sativa* leaf extract.

S. No	Compound Name	Molecular Formula	Molecular Weight	Retention Time (Min)	Probability Index	Percent Area of Curve
1	9,12-Octadecadienoic acid (Z,Z)-	C_18_H_32_O_2_	280	76.9	55.92	16.48
2	Cyclononasiloxane, octadecamethyl-	C_18_H_54_O_9_Si_9_	666	110.08	22.90	23.76
3	n-Hexadecanoic acid	C_16_H_32_O_2_	256	71.51	72.04	3.76
4	Hexadecanoic acid, 2- hydroxy-1- (hydroxymethyl)ethyl	C_19_H_38_O_4_	330	90.53	61.55	2.69
5	Octadecanoic acid, 2- hydroxy-1- (hydroxymethyl)ethyl ester	C_21_H_42_O_4_	358	95.25	63.34	1.37
6	Octadecanamide	C_18_H_37_NO	283	87.36	87	0.78
7	Stigmasterol	C_29_H_48_O	412	111.90	71.04	0.38
8	Benzothiazole	C_7_H_5_NS	135	34.31	8.06	0.2
9	Ethyl iso-allocholate	C_26_H_44_O_5_	436	76.13	7.51	0.15
10	Octacosane	C_28_H_58_	394	84.39	5.01	0.3

**Table 2:** A comparative study on the effects of *Lactuca sativa* leaf powder and omeprazole in the treatment of ulcers in Wistar rats.

Groups	Treatment	Ulcer Area (mm^2^)	% of Inhibition	Mucus Weight	pH
1	Normal control	0.00	0.00	2.8 ± 0.11	3.61 ± 0.09
2	Ulcer Control	611 ± 32	NA	0.95 ± 0.2	3.61 ± 0.21
3	Omeprazole	90 ± 3.5	85 ± 1.9	1.45 ± 0.3	6.54 ± 0.09
4	LPL	90.5 ± 10.58	85.18 ± 2.71	3.14 ± 0.28	7.01 ± 0.34

Each value is the mean of five batches (n = 5) with standard deviation. LPL: Leaf powder of Lactuca sativa.

## Data Availability

The data used to support the findings of this study are included within the article.

## References

[1] Wallace C.T., Bailey R.L., Blumberg J.B., Freeman B.B., Oliver Chen C.Y., Crowe-White K.M., Drewnowski A., Hooshmand S., Johnson E., Lewis R. (2020). Fruits, vegetables, and health: A comprehensive narrative, umbrella review of the science and recommendations for enhanced public policy to improve intake. Crit. Rev. Food Sci. Nutr..

[2] Noumedem J.A.K., Djeussi D.E., Hritcu L., Mihasan M., Kuete V., Victor K. (2017). Chapter 20—Lactuca sativa. Medicinal Spices and Vegetables from Africa.

[3] Kim H.D., Hong K.B., Dong O.N., Suh H.J. (2017). Sleep-inducing effect of lettuce (*Lactuca sativa*) varieties on pentobarbital-induced sleep. Food Sci. Biotechnol..

[4] Gopal S.S., Lakshmi M.J., Sharavana G., Sathaiah G., Sreerama Y.N., Baskaran V. (2017). Lactucaxanthin—A potential anti-diabetic carotenoid from lettuce (*Lactuca sativa*) inhibits α-amylase and α-glucosidase activity in vitro and in diabetic rats. Food Funct..

[5] Naseem S., Ismail H. (2022). In vitro and in vivo evaluations of antioxidative, anti-Alzheimer, antidiabetic and anticancer potentials of hydroponically and soil grown *Lactuca sativa*. BMC Complement. Med. Ther..

[6] Cheng D.M., Pogrebnyak N., Kuhn P., Krueger C.G., Johnson W.D., Raskin I. (2014). Development and phytochemical characterization of high polyphenol red lettuce with anti-diabetic properties. PLoS ONE.

[7] Hassan I.A., Basahi J.M., Ismail I.M. (2013). Gas exchange, chlorophyll fluorescence and antioxidants as bioindicators of airborne heavy metal pollution in Jeddah, Saudi Arabia. Curr. World Environ..

[8] Sanpinit S., Chonsut P., Punsawad C., Wetchakul P. (2022). Gastroprotective and Antioxidative Effects of the Traditional Thai Polyherbal Formula Phy-Blica-D against Ethanol-Induced Gastric Ulcers in Rats. Nutrients.

[9] Rahamat U.S., Sivakumar S.M., Raghad H.A., Rawan H.A., Nouf F.A., Khadijah M.W., Fayha N.A., Alshammari M.H., Fai M.A., ur Rehman Z. (2022). Spectral characterization of the bioactive principles and antibacterial properties of a cold methanolic extract of Olea europaea from the Hail region of Saudi Arabia, Arab. J. Chem..

[10] Rahamat U.S., Sivakumar S.M., Bader H., Ahmed A., Afnan A.A., Amr S.A., Amr S.A.L., Marwa H.A., Humera B., Mohd A.H. (2022). Bioactive principles, anti-diabetic, and anti-ulcer activities of *Ducrosia anethifolia Boiss* leaves from the Hail region, Saudi Arabia. Arab. J. Chem..

[11] Saad S.A., Sivakumar S.M., Muhammad H.S., Mohammed A.B., Osama A.M., Saeed A., Hafiz A.M., Santhosh J.M., ur Rehman Z., Alam M.d.S. (2022). Potential bioactive secondary metabolites of Actinomycetes sp. isolated from rocky soils of the heritage village Rijal Alma, Saudi Arabia. Arab. J. Chem..

[12] Al-Wajeeh N.S., Hajerezaie M., Noor S.M., Halabi M.F., Al-Henhena N., Azizan A.H.S., Kamran S., Hassandarvish A.N.S., Ali H.M. (2016). The gastro protective effects of cibotium barometz hair on ethanol-induced gastric ulcer in Sprague-Dawley rats. BMC Vet. Res..

[13] Sabiu S., Garuba T., Sunmonu T., Emmanuel A., Abdulhakeem S., Ismaila N., Abdulazeez B. (2015). Indomethacin-induced gastric ulceration in rats: Protective roles of Spondias mombin and Ficus exasperate. Toxicol. Rep..

[14] Njar V.C., Adesanwo J.K., Raji Y. (1995). Methyl angolensate: The antiulcer agent of the stem bark of *Entandrophragma angolense*. Planta Med..

[15] Tan P.V., Nyasse B., Dimo T., Mezui C. (2002). Gastric cytoprotective anti-ulcer effects of the leaf methanol extract of *Ocimum suave* (Lamiaceae) in rats. J. Ethnopharmacol..

[16] Unissa R., Moni S.S., Banu H., Alrahef S.S., Alrahef S.S., AlenezI T.K., Abdallah M.H., Abu Lila A.S., EL-Horany H., Abouzied A.S. (2022). Anti-ulcer properties, cytokines, and apoptosis regulatory effects of *Olea europaea* leaves from Hail Province, Saudi Arabia. Not. Bot. Horti Agrobot. Cluj-Napoca.

[17] Ülger T.G., Ayşe N.S., Onur C., Funda P.C. (2018). Chapter 2—Role of Vegetables in Human Nutrition and Disease Prevention. Vegetables—Importance of Quality Vegetables to Human Health.

[18] Hanin N.M., Abdurazag A.A. (2020). *Lactuca sativa* stems as the source of bioactive compounds as well as the leaves. J. Pharm. Pharmacol..

[19] Emil N.S., Sara V., Marcello I., Stefania G. (2021). Chemical characterization by GC/MS analysis of *Lactuca tatarica* (L.) C.A.Mey. aerial parts and seeds. Nat. Prod. Res..

[20] Chen Z.Y., Chan P.T., Kwan K.Y., Zhang A. (1997). Reassessment of the antioxidant activity of conjugated linoleic acids. J. Amer. Oil. Chem. Soc..

[21] Fagali N., Catalá A. (2008). Antioxidant activity of conjugated linoleic acid isomers, linoleic acid and its methyl ester determined by photoemission and DPPH techniques. Biophys. Chem..

[22] Den Hartigh L.J. (2019). Conjugated linoleic acid effects on cancer, obesity, and atherosclerosis: A review of pre-clinical and human trials with current perspectives. Nutrients.

[23] Sina N., Dagfinn A., Joseph B., Sara M., Masoomeh A., Omid S. (2011). Dietary intake and biomarkers of alpha linolenic acid and risk of all cause, cardiovascular, and cancer mortality: Systematic review and dose-response meta-analysis of cohort studies. BMJ.

[24] Watanabe Y., Tatsuno I. (2021). Omega-3 polyunsaturated fatty acids for cardiovascular diseases: Present, past and future. Expert Rev. Clin. Pharmacol..

[25] Guasch-Ferré M., Li Y., Walter C.W., Qi S., Laura S., Salas-Salvadó J., Martínez-González M.A., Meir J.S., Hu F.B. (2022). Consumption of olive oil and risk of total and cause-specific mortality among U.S. Adults. J. Am. Coll. Cardiol..

[26] Yang Q., Cao W., Zhou X., Cao W., Xie Y., Wang S. (2014). Anti-thrombotic effects of α-linolenic acid isolated from *Zanthoxylum bungeanum* maxim seeds. BMC Complement Altern. Med..

[27] Brzosko S., De Curtis A., Murzilli S., de Gaetano G., Donati M.B., Iacoviello L. (2002). Effect of extra virgin olive oil on experimental thrombosis and primary hemostasis in rats. Nutr. Metab. Cardiovasc. Dis..

[28] Ni Luh S. (2016). Identification of the Substance Bioactive Leaf Extract *Piper caninum* Potential as Botanical Pesticides. Int. J. Pure App. Biosci..

[29] Aparna V., Dileep K.V., Mandal P.K., Karthe P., Sadasivan C., Haridas M. (2012). Anti-inflammatory property of n-hexadecanoic acid: Structural evidence and kinetic assessment. Chem. Biol. Drug Des..

[30] Bakar K., Mohamad H., Latip J., Tan H.S., Herng G.M. (2017). Fatty acids compositions of Sargassum granuliferum and Dictyota dichotoma and their anti-fouling activities. J. Sustain. Sci. Manag..

[31] Bubenchikov R.A., Korableva T.V., Pozdnyakova T.A., Kuleshova E.S. (2020). The study of the Fatty acid composition of compass Lettuce (*Lactuca serriola* L.). Res. J. Pharm. Technol..

[32] Zhu S., Jiao W., Xu X., Hou L., Li H., Shao J., Zhang X., Wang R., Kong D. (2021). Palmitic acid inhibits prostate cancer cell proliferation and metastasis by suppressing the PI3K/Akt pathway. Life Sci..

[33] Giancarlo C.V., Carlimar O.M., Solymar M., Christian M.G., René García D.V., Néstor M.C., Sanabria-Ríos D.J. (2021). Antibacterial fatty acids: An update of possible mechanisms of action and implications in the development of the next-generation of antibacterial agents. Prog. Lipid Res..

[34] Havlicekova Z., Jesenak M., Banovcin P., Milan K. (2015). Beta-palmitate—A natural component of human milk in supplemental milk formulas. Nutr. J..

[35] Litmanovitz I., Bar-Yoseph F., Lifshitz Y., Davidson K., Eliakim A., Regev H., Nemet D. (2014). Reduced crying in term infants fed high beta-palmitate formula: A double-blind randomized clinical trial. BMC Pediatr..

[36] Glycerol Monostearate. https://www.chefsteps.com/ingredients/glycerol-monostearate.

[37] Gabay O., Sanchez C., Salvat C., Chevy F., Breton M., Nourissat G., Wolf C.J., Berenbaum F. (2010). Stigmasterol: A phytosterol with potential anti-osteoarthritic properties. Osteoarthr. Cartil..

[38] Tovey F.I., Capanoglu D., Langley G.J., Herniman J.M., Bor S., Ozutemiz O., Hobsley M., Bardhan K.D., Linclau B. (2011). Dietary phytosterols protective against peptic ulceration. Gastroenterol. Res..

[39] Shokraei S., Khandouzi N., Sina Z., Nasrollahzadeh J. (2021). The acute effect of incorporating lettuce or watercress into a moderately high-fat meal on postprandial lipid, glycemic response, and plasma inflammatory cytokines in healthy young men: A randomized crossover trial. Lipids Health Dis..

